# Supplementary material for: Deletion of wfs1 Impairs Oligodendrocyte Precursor Cells Dorsal Distribution and Myelination Through the wfs1-hmgcs1 Axis in Zebrafish
Source: Biology (Basel). 2026 Aug 16;15(16):1402. doi: 10.3390/biology15161402 (PMC13510151; doi:10.3390/biology15161402)
Supplement: Supplementary file 1 [file biology-15-01402-s001.zip › File S1-Transcriptome sequencing data and differentially expressed genes (DEGs) in wfs1-- zebrafish at 5 dpf/1.GO/wfs1KOvsWT/GSEA/WT/METHIONINE_METABOLIC_PROCESS/METHIONINE_METABOLIC_PROCESS(GO_0006555).html]

Details for gene set METHIONINE\_METABOLIC\_PROCESS(GO:0006555)[GSEA]


|  || Dataset | deg.sample |
| Phenotype | sample.cls |
| Upregulated in class | WT |
| GeneSet | METHIONINE\_METABOLIC\_PROCESS(GO:0006555) |
| Enrichment Score (ES) | -0.48079735 |
| Normalized Enrichment Score (NES) | -1.8074955 |
| Nominal p-value | 0.0 |
| FDR q-value | 0.2516796 |
| FWER p-Value | 0.659 |
Table: GSEA Results Summary

  

Fig 1: Enrichment plot: METHIONINE\_METABOLIC\_PROCESS(GO:0006555)
  
Profile of the Running ES Score & Positions of GeneSet Members on the Rank Ordered List

  

| PROBE | DESCRIPTION   (from dataset) | GENE SYMBOL | GENE\_TITLE | RANK IN GENE LIST | RANK METRIC SCORE | RUNNING ES | CORE ENRICHMENT || 1 | 404209 | cnr1 |  |  | 1658 | 1.563 | 0.0632 | No |
| 2 | 387530 | ahcy |  |  | 3139 | 1.004 | 0.0908 | No |
| 3 | 567547 | mthfr |  |  | 4740 | 0.713 | 0.0931 | No |
| 4 | 100002302 | mri1 |  |  | 6386 | 0.548 | 0.0819 | No |
| 5 | 378847 | mtr |  |  | 9320 | 0.346 | 0.0154 | No |
| 6 | 322228 | bhmt |  |  | 12711 | 0.154 | -0.0796 | No |
| 7 | 798918 | ahcyl1 |  |  | 16766 | -0.025 | -0.2049 | No |
| 8 | 393526 | mtap |  |  | 17330 | -0.047 | -0.2191 | No |
| 9 | 560667 | mtrr |  |  | 21227 | -0.227 | -0.3247 | No |
| 10 | 394240 | ahcyl2 |  |  | 21271 | -0.229 | -0.3092 | No |
| 11 | 795246 | cnr2 |  |  | 26207 | -0.548 | -0.4235 | No |
| 12 | 447941 | apip |  |  | 28033 | -0.747 | -0.4258 | Yes |
| 13 | 324079 | adi1 |  |  | 29660 | -1.115 | -0.3946 | Yes |
| 14 | 431773 | enoph1 |  |  | 31048 | -2.026 | -0.2888 | Yes |
| 15 | 791167 | zgc:158222 |  |  | 31768 | -4.275 | 0.0038 | Yes |
Table: GSEA details
[plain text format]

  

Fig 2: METHIONINE\_METABOLIC\_PROCESS(GO:0006555)
  
Blue-Pink O' Gram in the Space of the Analyzed GeneSet

  

Fig 3: METHIONINE\_METABOLIC\_PROCESS(GO:0006555): Random ES distribution
  
Gene set null distribution of ES for
 **METHIONINE\_METABOLIC\_PROCESS(GO:0006555)**
